# Insights into the Functions of LncRNAs in *Drosophila*

**DOI:** 10.3390/ijms20184646

**Published:** 2019-09-19

**Authors:** Keqin Li, Yuanliangzi Tian, Ya Yuan, Xiaolan Fan, Mingyao Yang, Zhi He, Deying Yang

**Affiliations:** 1 Institute of Animal Genetics and Breeding, Sichuan Agricultural University, Chengdu 611130, China; keqin.li.china@gmail.com (K.L.); tylz1996@outlook.com (Y.T.); yuanya145@163.com (Y.Y.); xiaolanfan@sicau.edu.cn (X.F.); yangmingyao@sicau.edu.cn (M.Y.); 2 Farm Animal Genetic Resources Exploration and Innovation Key Laboratory of Sichuan Province, Sichuan Agricultural University, Chengdu 611130, China; 3 College of Animal Science and Technology, Sichuan Agricultural University, Chengdu 611130, China; zhihe@sicau.edu.cn

**Keywords:** long non-coding RNAs, mechanism, *Drosophila*, transcriptome, biological function

## Abstract

Long non-coding RNAs (lncRNAs) are a class of non-coding RNAs longer than 200 nucleotides (nt). LncRNAs have high spatiotemporal specificity, and secondary structures have been preserved throughout evolution. They have been implicated in a range of biological processes and diseases and are emerging as key regulators of gene expression at the epigenetic, transcriptional, and post-transcriptional levels. Comparative analyses of lncRNA functions among multiple organisms have suggested that some of their mechanisms seem to be conserved. Transcriptome studies have found that some *Drosophila* lncRNAs have highly specific expression patterns in embryos, nerves, and gonads. In vivo studies of lncRNAs have revealed that dysregulated expression of lncRNAs in *Drosophila* may result in impaired embryo development, impaired neurological and gonadal functions, and poor stress resistance. In this review, we summarize the epigenetic, transcriptional, and post-transcriptional mechanisms of lncRNAs and mainly focus on recent insights into the transcriptome studies and biological functions of lncRNAs in *Drosophila*.

## 1. Introduction

Long non-coding RNAs (lncRNAs) have been defined as RNA transcripts that are longer than 200 nucleotides and lack a significant open reading frame. The majority of lncRNAs are transcribed by RNA polymerase II (Pol II) and have a 3′ polyadenylation, 5′ cap characteristic [1], with an average length of 1 kb [2]. Based on their positions relative to neighbouring genes, lncRNAs can be classified as divergent, convergent, intergenic, overlapping, enhancer, intronic, and microRNA (miRNA) host RNAs [3]. In contrast to protein-coding messenger RNAs (mRNAs) that are trafficked to the cytoplasm for translation [4], lncRNAs are predominantly localized in the nucleus [5]. They have highly abundant transcripts in many organisms, such as humans (167,150), mice (130,558), *Drosophila melanogaster* (54,818), and *Caenorhabditis elegans* (3269) [6]. A wide range of biological processes are under the control of lncRNAs, such as X-chromosome silencing, transcriptional activation, transcriptional interference, genetic imprinting, chromosome modification, and nuclear transport [7]. Therefore, lncRNAs occupy an irreplaceable position in the process of organismal growth, development, senescence, and death [8].

*D. melanogaster*, a well-established model organism, has the advantage of combining lower genetic redundancy with complex behaviour [9]. The time course of flies’ embryonic development begins at fertilization, and it takes about a day for an embryo to hatch out of the egg shell to become a first instar larva. The larva then passes through three stages separated by moults, and at the end of the third instar it pupates. Eventually, about nine days after fertilization, an adult fly emerges. Females can lay about 800 eggs in their lifetime. Due to the short generation time, high fecundity, and the subsequent low maintenance costs, *Drosophila* is considered one of the most useful model organisms. The fly genome has been extensively studied and fully sequenced with a wide range of genetic tools; gene-specific knockdown and mutant lines are readily available, including P-element mediated mutagenesis and the GAL4/UAS system, which allow for disrupting endogenous gene expression and introducing foreign genes in a temporal and tissue-specific manner, respectively. These tools have been applied to decipher the code of numerous fundamental biological processes, including embryonic development, neurological function, signal transduction, and behavioural patterns [9]. In addition, up to approximately 77% of human disease-related genes have functional *Drosophila* homologs, and many molecular mechanisms are conserved between species [10]. Based on these striking genetic similarities, the use of flies has been extended from basic research to modelling human disease models, and they have been used to identify novel pathogenic genes or to screen effective therapeutic drugs. For example, the *Drosophila* genome contains genes that encode orthologs of several major proteins involved in Alzheimer’s disease (AD) pathogenesis, and *Drosophila* AD models exhibit various easily visible and quantifiable phenotypes such as eye degeneration and learning and memory defects, which make it suitable for in vivo genetic screening. Based on these models, several biochemical processes have been found to be involved in mediating the toxic effects of amyloid β-peptide 42 (Aβ42), which is the major component of amyloid plaques in AD brains [11].

**Figure 1:**
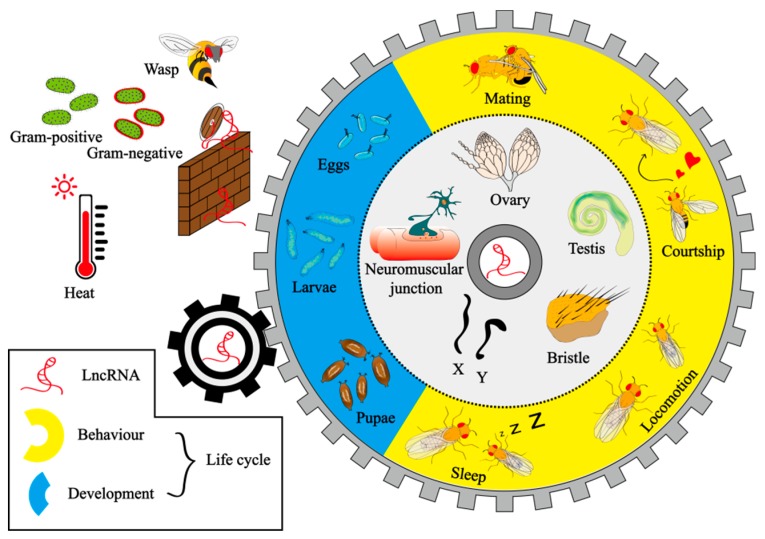
The biological function of long non-coding RNAs (lncRNAs) in *Drosophila*. LncRNAs regulate development (embryo, organ, and neuromuscular junction), behaviour (sleep, locomotion, courtship, and mating), sex determination, and dosage compensation in *Drosophila*. LncRNAs can also protect fruit flies from some stressors, such as heat and infection by bacteria and wasps.

Previous studies on lncRNAs have mainly focused on vertebrates such as mice and humans. With the employment of multiple technologies, experimental biologists have discovered a variety of lncRNA regulatory mechanisms in *Drosophila* in recent years, some of which are similar to those in humans and other mammalians. For example, in dosage compensation, both the mammalian lncRNA-Xist and lncRNA-roX in fruit flies can act to modulate the expression of genes on the sex chromosome by binding to a transcription modifier or chromatin modifier, and this similar mechanism of action could be due to conserved zinc-finger motifs in their sequences [10]. The mechanism of lncRNA-7SK in *Drosophila*, which is almost identical to that of its homolog in humans, may also result from the structural identity of the 7SK sequence in several regions between flies and humans [12]. LncRNAs in *Drosophila* have emerged as crucial regulators of organ development [13], neurological function [14,15], gonadal function [16], and stress response [17,18]. Here, we will briefly review the epigenetic, transcriptional, and post-transcriptional mechanisms of lncRNAs and then summarize their role in *Drosophila* with a particular emphasis on experimentally verified biological functions (Figure 1).

## 2. Conserved Secondary Structure Could Account for Functional Similarities

Despite the primary sequence of lncRNAs being weakly conserved across species, this does not imbue a lack of function. The RNA structure of lncRNAs appears to be the main functional unit and evolutionary constraint [19]. In zebrafish, the developmental phenotype defects following functional inactivation of conserved lncRNA-cyrano can be rescued by an orthologous gene from either mice or humans, although the human or mouse orthologs contain only very small areas of highly conserved primary nucleotide sequence [20]. Furthermore, introducing this nucleotide region into the heterologous RNA fails to rescue those abnormal phenotypes, indicating that it is more likely to be the higher-order structure of a lncRNA that is conserved and has complementary function rather than its primary sequence [21,22]. The RNA structure within the conserved repeats of lncRNA-Xist contains two loops, linked by AU-rich spacers whose sequence is not conserved between humans and mice [23]. Modifications to the sequence and the length of the linker sequence exhibit inability to affect recruitment of certain proteins which induce X-chromosome inactivation. However, deletion of a conserved 5′ element of lncRNA-Xist containing the highly conserved repeat units could completely abolish the silencing activity [23]. Therefore certain conserved secondary structures originated from the structural motif might be crucial for the function of lncRNAs.

## 3. Three Main Mechanisms of LncRNAs

**Figure 2:**
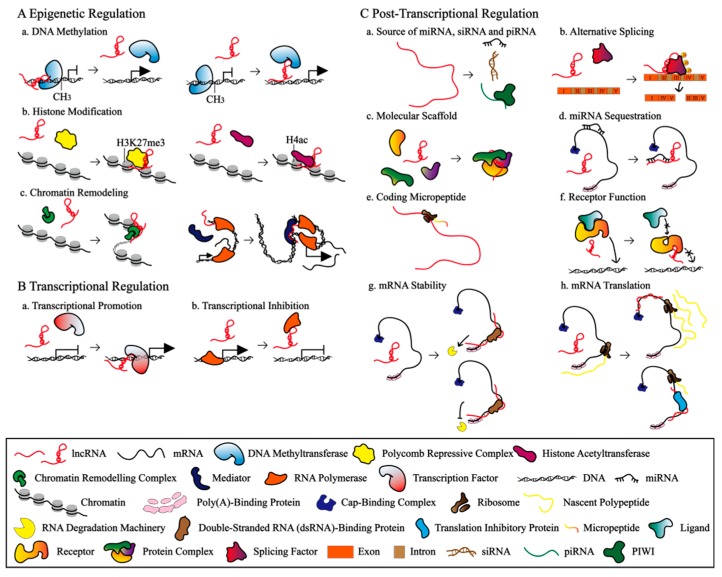
The epigenetic, transcriptional, and post-transcriptional mechanisms of lncRNAs. (**A**) Role of lncRNAs in the epigenetic regulation. (**a**) DNA methylation [25,26]. (**b**) Histone modification [27,28]. (**c**) Chromatin remodelling [29,30]. (**B**) Role of lncRNAs in transcriptional regulation. (**a**,**b**) LncRNAs regulate the binding of transcription factors to target genes [31,32]. (**C**) Role of lncRNAs in post-transcriptional regulation. (**a**) LncRNAs can serve as precursors of miRNAs, small interfering RNAs (siRNAs), or piwi-interacting RNAs (piRNAs) [33,34,35]. (**b**) LncRNAs can regulate alternative splicing [36]. (**c**) LncRNAs, such as metastasis-associated lung adenocarcinoma transcript 1 (MALAT1), can act as molecular scaffolds, allowing the assembly of protein complexes [37]. (**d**) LncRNAs control mRNA levels by functioning as competing endogenous RNAs (ceRNAs) [38]. (**e**) Some lncRNAs may encode micropeptides [39,40]. (**f**) LncRNAs may block the activity of protein receptors [41]. (**g**,**h**) LncRNAs can directly regulate the stability and translation of mRNAs [42,43,44,45].

The preservation of RNA secondary structures and even more restricted expression patterns of several lncRNAs contribute to the complex regulatory networks behind them. Serving as signals, decoys, guides, and scaffolds [24], lncRNAs take part in the regulation of gene expression at the epigenetic, transcriptional, and post-transcriptional levels (Figure 2).

### 3.1. Epigenetic Regulation

Regulation of gene expression at the epigenetic level is a stable alteration which could be heritable in the short term but does not involve mutations of the DNA itself [46]. Epigenetic regulation plays a vital role in the development of organisms and tumorigenesis [3], the mechanisms of which include DNA methylation, histone modification, and chromatin remodelling [47]. Nuclear-located lncRNAs mediate the level of DNA methylation and histone modification and accessibility of chromatin by recruiting or sequestering epigenetic modifiers such as DNA methyltransferase, histone deacetylase, and chromatin-modifying complex. In humans and mice, an antisense (AS) lncRNA-Pcdhα-AS is extended through the sense promoter of protocadherin (*Pcdh*), leading to DNA demethylation of the CCCTC-binding protein (CTCF) binding sites proximal to each promoter. Demethylation-dependent CTCF binding to both promoters facilitated cohesin-mediated DNA looping with a distal enhancer (*HS5-1*), locking in the transcriptional state of the chosen *Pcdhα* gene [26] (Figure 2Aa). LncRNA-IFI6 could inhibit the initial transcription of *interferon-inducible protein 6* (*IFI6*) as a response to hepatitis C virus (HCV) infection by regulating *IFI6* promoter histone modification of H3K4me3 and H3K27me3 in human cells [28] (Figure 2Ab). In mouse myogenic cells, linc-RAM regulated nucleosome positioning and expression of myogenic genes by directly binding to the master transcriptional regulatory factor of myogenic differentiation (MyoD), which in turn recruited the chromatin remodelling complex SWItch/Sucrose NonFermentable (SWI/SNF) [29] (Figure 2Ac).

### 3.2. Transcriptional Regulation

LncRNAs could regulate transcription in several ways: (1) lncRNAs can promote transcriptional activity as enhancer RNAs or by binding to a protein complex with enhancer activity; (2) DNA-binding protein and RNA polymerase can be recruited to a gene by nascent or dissociative lncRNAs *in cis* or *in trans*, respectively; and (3) lncRNA can inhibit the binding of a transcriptional regulatory factor by acting as a “decoy” or repress its activity by direct active-site occlusion or allosteric effects [46]. LncRNA-MeXis interacted with and guided promoter binding of the transcriptional coactivator DEAD-box helicase 17 (DDX17), regulating the expression of cholesterol homeostasis-related gene *ATP binding cassette transporter A1* (*Abca1*) in mouse bone marrow cells [31] (Figure 2Ba). LncRNA-a3 is associated with the human mediator complex that promotes the assembly of transcription factors and Pol II on target mRNA promoter sites to establish chromosomal loop structures. This complex promoted transcriptional activation by further phosphorylating histone H3S10 [48] (Figure 2Ba). In addition, lncRNA-NRON was conserved between mice and humans and it could repress nuclear trafficking of the dephosphorylated transcription factor nuclear factor of activated T cells (NFAT) by interacting with the importin-beta superfamily [49] (Figure 2Bb).

### 3.3. Post-Transcriptional Regulation

LncRNAs are implicated in the stability and translation of mRNAs, pre-mRNA splicing, protein activities, and precursors of miRNAs and siRNAs. In addition, they can encode some polypeptides and serve as miRNA sponges in both a sequence-dependent and sequence-independent manner [50]. Some lncRNAs, such as lncRNA-H19, could act as precursors of miRNAs. During skeletal muscle differentiation and regeneration in mice, lncRNA-H19 encoded two conserved microRNAs, miR-675–3p and miR-675–5, which could function by directly downregulating the anti-differentiation Smad transcription factor [51] (Figure 2Ca). Another lncRNA, lncRNA-MEG3-4, served as a ceRNA and modulated the mRNA abundance of *IL-1β* by competitively binding to miR-138 in mouse lungs [52] (Figure 2Cd). Other evidence suggests that lncRNAs can directly bind to mRNAs to mediate the regulation of mRNA stability or translation. Glucose deprivation-induced expression of lncRNA-MACC1-AS1 promoted *MET transcriptional regulator MACC1* (*MACC1*) mRNA stability via the AMP-activated protein kinase (AMPK)/Lin28 pathway in human cells [42] (Figure 2Cg). LncRNA-AdipoQ-AS transferred from the nucleus to the cytoplasm and inhibited adipogenic differentiation by forming a sense/antisense RNA duplex to prevent the translation of mouse *adiponectin* (*AdipoQ*) mRNA [45] (Figure 2Ch). In addition, the nuclear-retained lncRNA-MALAT1 regulated alternative splicing by modulating serine/arginine (SR) splicing factor phosphorylation in both mice and humans [36] (Figure 2Cb). Furthermore, lncRNA-DINO, which was conserved in mice and humans, might promote p53 protein stabilization through its interaction with p53, which resulted in the amplification of DNA damage signalling [53]. A few lncRNAs, such as lincRNA-00961, could encode polypeptides. LincRNA-00961 encoded small regulatory polypeptide of amino acid response (SPAR), which was conserved between humans and mice, localized to the late endosome/lysosome, and could interact with the lysosomal vacuolar ATPase (v-ATPase) to negatively regulate mechanistic target of rapamycin complex 1 (mTORC1) activation [40] (Figure 2Ce).

## 4. LncRNAs Mainly Associated with *Drosophila* Gonads and Development by Transcriptomics Analysis

With commonly used measures of lncRNA microarrays, RNA sequencing (RNA-seq), and bioinformatics analysis, lncRNAs found in *Drosophila* accounted for an abundant and similar proportion of the entire genome to that in humans, comprising 13.5% and 12.3% of annotated genes, respectively [10]. In *Drosophila*, many of these lncRNAs were found to have a more specific expression pattern in gonads and during development, which might reveal the important functions of lncRNAs.

### 4.1. Gonads

In the gonads, lncRNAs had different distribution locations. Many lncRNAs (563 or 30%) had peak expression in the testes, and 125 of these lncRNAs were detectable in only the testes [54]. The transcriptomes of three different parts of the wild-type testis were analysed, including the apical region, the middle region, and the basal region [55]. A total of 203 lncRNAs showed significant differences between the apical and basal regions of the testis [55]. Most of the differentially expressed lncRNAs had significantly higher transcript levels in the basal regions (containing elongated post-meiotic cysts with spermatids) than in the other regions, indicating a post-meiotic function for most of the tested lncRNAs [55]. A similar result has been reported earlier, and showed that the majority of testis-specific lncRNAs were strictly expressed in the meiotic and post-meiotic region of the testis [56]. The observation of *Drosophila* lncRNAs with differential expression pattern in testis is consistent with studies in humans and mice [57,58]. In the mouse germline, the unique expression of lncRNAs might be attributed to the change of epigenetic modifications. Potentially functional lncRNAs show significantly lower promoter CpG methylation levels in the germline [57], and more up-regulated lncRNAs in mouse testis possess H3K4me3 marks on their promoters [59]. Promoters of these lncRNAs are on average more conserved than those of protein-coding genes and are associated with chromatin marks [59]. In *Drosophila*, knockdown of some testis-specific lncRNAs frequently resulted in late spermatogenesis defects, which may indicate their regulatory function at spermatogenesis and male fertility [56].

### 4.2. Development

During the 27 developmental processes of metamorphosis in *Drosophila*, lncRNA expression profiling revealed that they had more temporally restricted expression patterns than those of the protein-coding genes [60]. Although the expression patterns were strictly restricted, 21% and 42% of lncRNAs were significantly upregulated during the late embryonic stage and the larval stage, respectively [60]. At this critical moment in the initial stage of metamorphosis, the considerable upregulation of lncRNAs signified that lncRNA enrichment in development could be important for transformation and organogenesis [60]. Another study demonstrated that the sequence and expression specificity of non-coding RNA promoters were evolutionarily conserved in five *Drosophila* strains and that a substantial proportion of lncRNAs might be related to embryonic development [61]. RNA-seq of three different parts among multiple developmental stages of embryos suggested that: (1) most lncRNAs had dynamic expression patterns; (2) a set of lncRNAs showed significant Gene Ontology (GO) term enrichments, which were mainly related to segment specification; and (3) the majority of the 30 tested lncRNAs gave a specific and even restricted expression atlas [62]. Moreover, with massively parallel droplet-based single-cell sequencing of *Drosophila* stage 6 embryos and a novel spatial mapping strategy, a virtual embryo with single-cell transcriptome resolution has been reconstructed recently. Based on this virtual embryo, an interactive “*Drosophila*-Virtual-Expression-eXplorer” (DVEX) database that allows for generation of virtual in situ hybridizations and computation of gene expression gradients has been built, through which ~40 lncRNAs were identified to play an unrecognized role in early embryonic patterning and development, and their expression patterns were also predicted and partially confirmed [63].

### 4.3. Ageing and Neurogenesis

Likewise, several lncRNAs have shown tissue and temporal patterns of expression during ageing and neurogenesis by other studies. A portrait of the transcriptome of lifespan extension by dietary restriction (DR) recognized differentially expressed profiles of lncRNAs [64]. The GO and Kyoto Encyclopedia of Genes and Genomes (KEGG) analysis showed that the targets of these lncRNAs were enriched in ageing-related pathways, such as the forkhead box O (FoxO) signalling pathway [64]. A single-cell atlas of adult fly brains had been built and uncovered the dynamic changes in the neuronal and glial cells during ageing. LncRNA-hsrω and lncRNA-CR34335 were predicted as two of the most important genes in cellular ageing through a random forest regression model [65]. Recently, a set of 13 lncRNAs were found to be specifically expressed in neuroglial lineages during embryonic neurogenesis in *Drosophila*. These lncRNAs were regulated in a tissue-specific manner and exhibited a spatiotemporal expression pattern during neurogenesis with exquisite specificity, indicating that neurogenic lncRNAs can mark specific neuroglial subsets and that some lncRNAs might play a key role in neurogenesis [66].

## 5. Biological Functions of LncRNAs in *Drosophila*

In the last decade, *Drosophila* lncRNA functions in vivo have been exposed constantly (Table 1). *Drosophila* lncRNAs act throughout diverse biological processes, including the development of embryos [12,13,67,68,69,70,71], bristles [72,73], gonadal cells [16,74], and neuromuscular junctions [15,75] (Figure 1). The mechanisms of these lncRNAs act in a similar way to those in other organisms, including the regulation of the activity of enzymes or the status of response elements, interaction with certain proteins, transcriptional interference by overlapping with target genes, and the production of miRNAs.

### 5.1. Embryonic Development

Since the expression profiles of the majority of tissue- and temporal-specific lncRNAs are important markers for the developmental state [4], it is not surprising that lncRNAs are essential to developmental progress. During embryonic development, some lncRNAs are found to be regulators of nearby protein-coding genes [69], and others affect the status of the response element [13,71] and sequester the key kinase [12].

#### 5.1.1. LncRNA-bxd

The promoter/enhancer regions of the Hox gene clusters have been extensively studied for their transcriptional activities. Multiple non-coding transcripts are generated from these regions, including bithorax complex non-coding RNA transcripts [82]. These lncRNAs are transcribed only from the active domains of the *Drosophila* bithorax complex, and first appear in the blastoderm stage. LncRNA-bxd, a 23-kb transcript from the *bithoraxoid* (*bxd*) domain of the bithorax complex, may play a role in regulating the transcription of *ultrabithorax* by establishing active domains across the *bxd* polycomb response element in early embryos [13].

#### 5.1.2. LncRNA-lincX

LncRNA-lincX is associated with the activation of *Sex combs reduced* (*Scr*) *in cis* [67]. It is transcribed from identified *cis*-regulatory sequences of the Hox gene *Scr*. Transcription of lncRNA-lincX precedes and fully overlaps the expression of *Scr*. Ectopic overexpression of lncRNA-lincX suggests transcription through the lncRNA-lincX locus, but not the RNA itself, may facilitate initiation of *Scr* in the early embryo *in cis*. Moreover, the regulation of *Scr* by lncRNA-lincX appears to be related to the transvection effect [67].

#### 5.1.3. LncRNA-acal

Embryonic dorsal closure (DC) is an ideal model for the regulation and manner of cell shape changes. Nuclear-retained lncRNA-acal is identified and characterized as a novel negative dorsal closure regulator, the mutation of which results in partially penetrant DC defects due to the over-activation of Jun N-terminal kinase (JNK) signalling. LncRNA-acal, which is expressed in the lateral epidermis, is regulated by raw and conserved pioneer proteins in diverse dipteran species. It shows genetic interaction with polycomb and negatively modulates the expression of scaffold protein connector of kinase to AP1 (Cka) and transcription factor anterior open (Aop) *in trans*, while these two proteins fine-tune JNK activation to the leading edge cells [68].

#### 5.1.4. LncRNA-ASTR

During embryogenesis, stable intronic sequence RNA-1 (sisRNA-1) represses lncRNA-ASTR with consequential effects on *regena* pre-mRNA expression [69]. LncRNA-ASTR is a *cis*-natural antisense transcript from the *regena* locus, and both are highly expressed in early embryos. Expression of lncRNA-ASTR shRNA results in a robust knockdown of ASTR with a significant decrease in the expression of *regena* pre-mRNA [69]. It is a link to sisRNAs and the expression of protein-coding genes, even though its mechanism is unclear.

#### 5.1.5. AAGAG Repeats RNAs

The forward and reverse strands of some large transcripts from the pericentromeric AAGAG repeats are crucial ingredients of the nuclear matrix and play important roles in genome maintenance, while the polypurine strands form the main proportion [70]. These AAGAG architectural RNAs (arcRNAs) are essential for viability and normal development since global or tissue-specific RNA interference (RNAi) of these transcripts would disrupt nuclear chromatin organization and even lead to lethality at the embryonic or late larval and pupal stages [70].

#### 5.1.6. LncRNA-vg-PRE/TRE

Some promoter-associated transcripts switch between the forward and reverse directions and thereby regulate the enhancer activity [83]. The *Drosophila vestigial* polycomb/trithorax response element (PRE/TRE) is transcribed in a bidirectional and developmentally regulated manner, yielding a pair of forward and reverse non-coding transcripts named here as lncRNA-vg-PRE/TRE. This pair of lncRNAs switches the status of the PRE/TRE between silenced and active. Only the reverse strand could bind to polycomb repressor complex 2 (PRC2) in vivo, although both of these lncRNAs inhibit PRC2 histone methyltransferase activity in vitro. Overexpression of the reverse strand could segregate PRC2 from chromatin, inhibit its enzymatic activity and further activate endogenous PRE/TRE. In contrast, the forward strand facilitates PRE/TRE pairing and the repression of endogenous *vestigial* mRNA. Dynamic and developmental switching of PRE/TRE properties by this forward and reverse lncRNAs contributes to the maintenance of cell identities during development [71].

#### 5.1.7. LncRNA-7SK

In humans, the positive transcription elongation factor b (P-TEFb) plays a key role in the regulation of transcription, and this is achieved by a complex regulatory system that controls the sequestration and release of P-TEFb from an inhibitory complex, the 7SK small nuclear ribonucleoprotein (7SK snRNP), which is built on the 7SK scaffolding RNA (h7SK). It contains two major components, the La-related protein (LARP7) and the double-stranded RNA-binding protein hexamethylene bisacetamide-induced protein 1/2 (HEXIM1/2), which interacts with and inhibits P-TEFb [12]. In *Drosophila* there exists a similar P-TEFb control system in which the *Drosophila* 7SK snRNP (d7SK snRNP) is also responsible for the release of P-TEFb through the action of the homolog of human HEXIM1/2 proteins (dHEXIM), and the main structural components of h7SK are also found in d7SK [12]. The beginning and end of the d7SK sequence are identical to those of the h7SK sequence, and it contains two AUCUG sequences separated by 8 nt that are exactly like those in h7SK [84], which might contribute to their similar mechanisms. In addition, both dHEXIM and dLARP7 (a homolog of human LARP7) are found to be essential for the growth and differentiation of tissues required during *Drosophila* development [12].

### 5.2. Neurodegenerative Disease

Neurodegenerative diseases such as AD, Parkinson’s disease (PD), and amyotrophic lateral sclerosis (ALS) have common cellular and molecular mechanisms, including the accumulation of protein aggregates [85]. Some of these aggregates are toxic to cells [86]. In *Drosophila*, two lncRNAs, lncRNA-hsrω [14] and lncRNA-CR18854 [15], are involved in a common pathway in Charcot–Marie–Tooth disease (CMT) and ALS pathogenesis. These may be helpful for gaining insight into the pathogenesis and therapy of neurological diseases.

#### 5.2.1. LncRNA-hsrω

LncRNA-hsrω, one of the most active genes after heat exposure, contributes to omega speckle formation and thermotolerance [17]. Distinct from its stress-responsive feature, lncRNA-hsrω also participates in the development of neuromuscular junctions. Neuron-specific and motor neuron-specific interference of lncRNA-hsrω damages locomotion, shortens the life span, and induces anatomical defects in the presynaptic terminals of motor neurons. In humans, the aggregation-prone heterogeneous nuclear ribonucleoprotein (hnRNP) human fused in sarcoma (hFUS) could aberrantly form immunoreactive inclusion bodies in a range of neurological diseases classified as FUS-proteinopathies, and its homolog *Drosophila* FUS (dFUS) is a hsrω-interacting protein in *Drosophila* [14]. A previous study shows that knockdown of lncRNA-hsrω strongly affects the expression and subcellular localization of dFUS. dFUS appears to mislocate and be largely present in the cytoplasm of the neurons with a lncRNA-hsrω deficiency [14]. Moreover, when hFUS is expressed in fly eyes, RNAi of lncRNA-hsrω could even lead to a removal of hFUS aggregates; thus, the toxicity of hFUS could be rescued by modulating lncRNA-hsrω expression, and this improvement partly depends on lysosomal-associated membrane protein 1 (LAMP1) [75]. These novel results reveal an evolutionarily conserved lncRNA-dependent mechanism to control FUS transcripts, and this may provide new ideas for further research of the pathomechanism of FUS-proteinopathies.

#### 5.2.2. LncRNA-CR18854

*FIG4* is one of the causative genes for CMT that affects both motor and sensory peripheral nerves [87]. Neuron-specific knockdown of the *Drosophila FIG4* (*dFIG4*) gene leads to the impaired locomotive abilities of adult flies and causes defective neuromuscular junctions, such as reduced synaptic branch length in presynaptic terminals of the motor neurons [15]. Nevertheless, lncRNA-CR18854 could rescue the rough eye phenotype and the loss-of-cone cell phenotype caused by eye imaginal disc-specific knockdown of *dFIG4*. In addition, mutation and knockdown of lncRNA-CR18854 partly suppress the enlarged lysosome phenotype induced by *dFIG4* deficiency in the fat body. Further genetic screening indicates a genetic interaction between lncRNA-CR18854 and *dFUS*, which is one of the pathogenic genes for ALS [15].

### 5.3. Behaviour

*Drosophila* has been well exploited to gain insights into the genetic basis of fly behaviour [88]. The location within the neural gene cluster of lncRNAs and the regulation of neural development-related protein by lncRNAs reflect a probable relationship between lncRNAs and behaviour. To date, several lncRNAs have been found to be functional in sleep [81], locomotion [76], courtship [79], and mating behaviour [77].

#### 5.3.1. LincRNA-yar

LincRNA-yar is located in the intergenic region between the *yellow* and *achaete* genes. The locus of lncRNA-yar, including its promoter, is conserved across *Drosophila* species, representing 40–60 million years of evolution [81]. In addition, the temporal expression patterns of lncRNA-yar are similar between *Drosophila virilis* and *Drosophila melanogaster*, suggesting that the transcriptional regulation of lncRNA-yar is conserved. Lacking lncRNA-yar causes no obvious defects in morphology or vitality. Nullisomy of lncRNA-yar causes a reduction and fragmentation in night time sleep time, with decreased sleep rebound following sleep deprivation [81]. According to the cytoplasmic localization of lincRNA-yar and its incapacity to affect transcription of the neighbouring genes, a possible link between lincRNA-yar and miRNAs has been investigated and about 33 miRNAs are found to match with its exons [81], indicating that lincRNA-yar may function as a sponge of miRNAs.

#### 5.3.2. LncRNA-CRG

LncRNA-CRG is involved in locomotor activity and climbing ability [76]. LncRNA-CRG shows relatively restricted expression in the central nervous system (CNS) from the embryonic to the adult stages. LncRNA-CRG is located downstream of *Ca^2+^/calmodulin-dependent protein kinase* (*CASK*), a behaviour-related coding gene, and partially overlaps with the 3′ UTR of *CASK*. The sequence of lncRNA-CRG is highly conserved across the 12 *Drosophila* species. The nullisomy of lncRNA-CRG exhibits a significant reduction in the abundance of the *CASK* transcript and protein. The defective phenotypes in climbing ability between lncRNA-CRG and the *CASK* mutant are similar. The defects of the lncRNA-CRG mutant could be rescued by *CASK* overexpression. Furthermore, lncRNA-CRG promotes *CASK* expression by recruiting Pol II to the *CASK* promoter [76].

#### 5.3.3. LncRNA-Sphinx

LncRNA-Sphinx has a lineage-specific expression pattern that is involved in regulating courtship behaviour [79]. The 5′ flanking region of the lncRNA-Sphinx gene is conserved across *Drosophila* species and could be expressed in the male accessory gland with promotion of the highly conserved segment. LncRNA-Sphinx signals are also caught in the brain, wing hairs, and leg bristles. Moreover, a putative lncRNA-Sphinx expression signal is identified in the brain antennal lobe and inner antennocerebral tract, suggesting that lncRNA-Sphinx might be involved in olfactory neuron-mediated regulation of male courtship behaviour. The lncRNA-Sphinx knockout mutation shows significantly upregulated gene categories related to accessory gland protein function and odour perception, revealing that it might be a negative regulator of its target genes [79].

#### 5.3.4. LncRNA-iab-8

The homeotic genes determine the posterior thorax and each abdominal segment of the fly, while genes affecting the more posterior segments repress the more anterior genes [89]. LncRNA-iab-8 is generated from the intergenic region between the homeotic *abd-A* and *Abd-B* genes and represses the expression of *abd-A* in the posterior CNS [77]. The lack of lncRNA-iab-8 shows ectopic expression of *abd-A* in the epidermis of the eighth abdominal segment. There are two mechanisms by which lncRNA-iab-8 represses *abd-A*, first through the production of miR-iab-8 (acting *in trans*) and second through transcriptional interference with the *abd-A* promoter (acting *in cis*). The most likely mechanism is that the 3′ end of lncRNA-iab-8 nascent strand overlaps with the *abd-A* promoter and sequesters RNA polymerase from the *abd-A* promoter. Knocking down lncRNA-iab-8 expression results in male and female sterility, which is independent of the problem with gametogenesis, gonads, or the external genitalia but is caused by a behavioural phenotype. The reason is that the male abdomen fails to bend, thereby preventing copulation with female flies, while eggs cannot pass through the oviduct, possibly because of a peristaltic wave disorder in female flies [77].

### 5.4. Gonads

In parallel with the recognition of differentially expressed genes in the gonad transcriptome, two lncRNAs have been demonstrated to affect gonadal cell development.

#### 5.4.1. LncRNA-msa

LncRNA-msa is another transcript from the *Drosophila* bithorax complex and shares much of its sequence and the same miR-iab-8 with lncRNA-iab-8. LncRNA-msa is essential for the development of the secondary cells of the *Drosophila* male accessory gland [16]. In secondary cells, lncRNA-msa acts primarily through miR-iab-8 coded in one of its introns. Deficiency of lncRNA-msa causes defects in secondary cell morphology and problems with male fertility, such as not generating long-term post-mating responses in his mate. In addition, the targets of lncRNA-msa probably are different from their targets in CNS [16].

#### 5.4.2. LncRNA-oskar

Otherwise, some of the well-known protein-coding genes may also have independent functions as lncRNAs [90]. In humans, distinct from the well-known roles of p53 protein in protecting the genome, *p53* mRNA is found to directly interact with the N-terminus of murine double minute 2 (MDM2) to prevent its E3 ubiquitin ligase activity [91]. Besides, *insulin receptor substrate 1* (*Irs1*) could encode protein IRS1, which is a major substrate and cytoplasmic docking protein for the insulin receptor (IR) and insulin-like growth factor receptor (IGF). However the *Irs1* mRNA could also function as a regulatory RNA and mediate the *retinoblastoma* (*Rb*) mRNA expression in human myoblasts through the complementary sequence in 5′ UTR of *Irs1* mRNA [92].

The *Drosophila* maternal effect gene *oskar* encodes the protein oskar and has distinct roles in germ line determination and posterior abdominal segment differentiation [93]. However, during early *Drosophila* oogenesis, *oskar* RNA plays an important role through a translation-independent mode that acts as lncRNAs [74]. Numerous reductions in *oskar* RNA levels show a sterile phenotype because of the early arrest of oogenesis. Moreover, expression of the *oskar* 3′UTR is sufficient to recover the egg-less defect of the RNA null mutant independent of protein. Previously, the localization of Staufen, an RNA-binding protein, within the oocyte is interdependent with that of *oskar* mRNA [94]. In the *oskar* null mutant, the Staufen protein fails to transport from the nurse cells into the oocyte. Expression of the *oskar* 3′ UTR alone is sufficient to restore Staufen accumulation in the oocyte. This reveals that the mutual interdependence of Staufen and *oskar* RNA in their localization during oogenesis is mediated by the interaction of Staufen with the *oskar* 3′ UTR [74]. Another possibility is that this non-coding function is mediated partly through sequestration of the translational regulator Bruno, which binds to Bruno response elements in its 3′ UTR [90].

### 5.5. Sex Determination and Dosage Compensation

The role of lncRNAs in sex determination has been widely studied. Due to the difference in the number of X-chromosome copies, a compensation pathway is required for genes located on X-chromosome to maintain a similar expression level. Sex determination and dosage compensation in *Drosophila* are implemented by the ratio of X-chromosomes to sets of autosomes [95]. The *Drosophila sex-lethal* (*sxl*) gene is the master regulator of these two processes and is regulated by several lncRNAs [80]. While in human, the genes on the X-chromosome in females are partially inactivated to achieve a similar level of expression to that in human males, and there also exist several lncRNAs engaging in this regulation [96].

#### 5.5.1. LncRNA-Sxl_Pe_-R1 and R2

Certain lncRNAs originate from two regions, R1 and R2, upstream of Sxl_Pe_ and named here as lncRNA-Sxl_Pe_-R1 and R2. To specify the female sex, the lncRNA-Sxl_Pe_-R1 and R2 show a dynamic developmental profile and activated Sxl_Pe_, which is the dose-sensitive establishment promoter of *Sxl*. Consistent with the timing of Sxl_Pe_ transcription, R2 AS is regulated by the X-chromosome counting genes, whereas the R1 transcripts are negative regulators of the activation of Sxl_Pe_. Ectopic expression of these lncRNAs also exhibits a change in the local chromatin marks and rescues effects of polycomb/trithorax group (PcG/trxG) mutations, affecting the timing and strength of Sxl_Pe_ transcription *in trans*. In addition, binding between the lncRNAs and the PcG/trxG results in chromatin alteration at the Sxl_Pe_ locus, and the interplay and regulatory network of lncRNA strands ultimately determine the consequences [80].

#### 5.5.2. LncRNA-roX1 and roX2

In *Drosophila*, lncRNAs are central in dosage compensation, and this is mediated by two non-coding RNAs, roX1 and roX2, which together with five proteins form the male-specific lethal (MSL) ribonucleoprotein complex [97]. Synchronous removal of lncRNA-roX1 and roX2 decrease X-chromosome localization of the MSL complex and cause later ectopic binding to autosomal sites and the chromo-centre. Global expression of the X-chromosome declines by 26% in the lncRNA-roX1 and roX2 male larvae mutant; moreover, this misregulation is similar in the lncRNA mutant and MSL protein deficiency [78]. Recently, another study reveals that lncRNA-roX1 and lncRNA-roX2 have partly separable functions in dosage compensation. In larvae, lncRNA-roX1 is the most abundant and the only variant present in the MSL complex when the complex is transmitted in mitosis. The loss of lncRNA-roX1 exhibits reduced expression of the genes on the X-chromosome, while the loss of lncRNA-roX2 leads to the MSL-independent upregulation of genes with male-biased testis-specific transcription [97]. In contrast to lncRNA-roX1, lncRNA-Xist is a lncRNA involved in the sex chromosome dose compensation pathway in humans. Like the roX genes, it can also coat the X-chromosome, where it regulates chromatin modification and thus affects the expression of specific target genes. However, lncRNA-Xist is expressed in females and regulates X-chromosome inactivation by promoting its initiation and stabilization [96]. Similar function with different sequences in these lncRNAs might be caused by conserved zinc-finger motifs [10]. These motifs have been identified in human and mouse genomes, and they are needed for lncRNAs involved in chromatin regulation to bind DNA and RNA [98].

### 5.6. Bristle Morphogenesis

The external sensory organ of *Drosophila*, the sensory bristle, originates from sensory organ precursor (SOP) cells after two asymmetric cell divisions (ACDs) [99]. It involves the development of neurons and the formation of sensory organs, and it is an effective model for studying ACD. The loss of bristle phenotypes could be caused by defects in SOP specification or survival or by cell fate transformations [100].

#### 5.6.1. LncRNA-bereft

LncRNA-bereft is regulated by the neural selector gene *cut* and further contributes to bristle morphogenesis [72]. Mutations of lncRNA-bereft result in the loss or malformation of a majority of the interommatidial bristles (IOBs) of the eye and missing of part of bristles of the head. LncRNA-bereft acts downstream of *cut* and *tramtrack*, and the expression levels of lncRNA-bereft are affected by *cut*, *tramtrack*, and *numb*. Furthermore, *cut* overexpression induces ectopic lncRNA-bereft expression in the peripheral nervous system (PNS) and the nonneuronal epidermis. Most of the IOB shafts are missing with abnormal socket morphology, implying that deficiency of bristle phenotypes in the lncRNA-bereft mutant might be attributed to the abnormal transformation of ACD [72].

#### 5.6.2. LncRNA-SMRG

Another bristle-related lncRNA with a more distinct mechanism has been reported recently. LncRNA-SMRG regulates *Drosophila* macrochaetes by antagonizing *scute* through E(spl)mβ [73]. This novel lncRNA is mainly distributed in the adult head and thorax and rarely in the abdomen. Highly conserved regions are spread across nearly the whole of the lncRNA-SMRG sequence by analysing 23 *Drosophila* species and houseflies, honeybees, mosquitoes, and beetles. The null mutant of lncRNA-SMRG exhibits more scutellar macrochaetes than the wild-type. Supernumerary scutellar macrochaetes trigger by elevated expression of proneural gene *scute* in the adult thorax while overexpression of lncRNA-SMRG or *scute* could rescue the supernumerary phenotype in the lncRNA-SMRG mutant. This antagonistic effect of lncRNA-SMRG on *scute* is mediated by E(spl)mβ. RNA immunoprecipitation (RIP) assay and chromatin immunoprecipitation (ChIP) assay indicate that lncRNA-SMRG negatively regulates *scute* expression by interacting with the repressive transcription factor E(spl)mβ and recruiting it to the *scute* promoter region [73].

### 5.7. Immunometabolism and Stress Resistance

#### 5.7.1. LincRNA-IBIN

LincRNA-IBIN is identified as the first lncRNA that connects immunity to metabolism in *Drosophila*. This lncRNA is highly induced during a Gram-positive bacterial infection. Further, expression of lincRNA-IBIN is also induced by Gram-negative bacteria in *Drosophila* adults and parasitoid wasps in *Drosophila* larvae [18]. This induction is dependent on the functional Toll and immune deficiency (Imd) pathway and the Osa-containing Brahma (BAP) complex. After infection, lincRNA-IBIN responds to nuclear factor-κB (NF-κB) signalling and the chromatin modelling Brahma complex and is specifically expressed in the fat body, haemocytes, and gut. The nuclear abundance of linc-IBIN suggests that its function may be in the regulation of gene expression, which is typical for lncRNAs, rather than to function as antimicrobial peptides (AMPs) [18]. Overexpressing lincRNA-IBIN induces the expression of Toll pathway-mediated genes, such as AMPs, and improved survival after infection. Otherwise, overexpression of lincRNA-IBIN in haemocytes increases haemocyte numbers, while global overexpression of lincRNA-IBIN elevates sugar levels in the haemolymph by enhancing the expression of genes that are important for glucose retrieval [18].

#### 5.7.2. LncRNA-hsrω

Distinct from neuromuscular function has been described; lncRNA-hsrω plays a vital role in thermotolerance in facing heat stress [17]. Upon temperature shock, the nullisomy, RNAi, or overexpression of lncRNA-hsrω imply lethality in most embryos and first- or third-instar larvae. Three-day-old null flies of lncRNA-hsrω have a poor prognosis after heat shock, while both down- and upregulation of lncRNA-hsrω restrain the reappearance of the lncRNA-hsrω-dependent nucleoplasmic omega speckles during recovery from heat shock. LncRNA-hsrω is responsible for the spatial restoration of key regulatory factors to their pre-stress nuclear targets in cells recovering from thermal stress. Failure of correct relocation to pre-stress chromosome sites results in restoration failure for normal developmental gene activity, and finally, organismal death [17].

In conclusion, serving as precursors of miRNAs and scaffolds of certain protein complex, as well as playing roles in recruiting certain proteins and transcriptional interference, lncRNAs act throughout the *Drosophila* life cycle. The function of lncRNAs covers development, behaviour, stress resistance, sex determination, and dosage compensation in *Drosophila*. Coupled with a completely sequenced genome, conservation of disease orthologs, and available genetic tools, *Drosophila* would be an ideal test tube for lncRNA studies that might be helpful to elucidate fundamental mechanisms of lncRNAs and their functions in a series of diseases and give new ideas to the identification of potential therapeutic targets. Also, since *Drosophila* is evolutionarily distant from humans, perhaps lncRNA research conducted in *Drosophila* may give us more inspiration from the perspective of evolution.

## 6. *Drosophila* LncRNA-related Databases

The application of the various transcriptomic approaches and modern computational approaches yielded a large number of lncRNA datasets, leading to the establishment of many lncRNA-related databases (Table 2). Utilizing these databases could facilitate the exploration of mechanisms of lncRNAs effectively. FlyAtlas 2 provided information for the expression of different transcripts of genes and sex-specific data for adult somatic tissues of *D. melanogaster* [101]. The *Drosophila* Interactions Database (DroID) was a comprehensive, integrated database for proteins, transcription factors, RNA, and gene interactions for *Drosophila* [102]. In addition, DVEX was an online resource that allowed for querying the single-cell expression atlas within a virtual embryo, and this high-resolution transcriptome map also provided an evolutionary comparison of gene expression patterns of two *Drosophila* species [63]. Other databases, such as CRISPRlnc and NPInter v3.0, collected validated CRISPR/Cas9 single guide RNAs (sgRNAs) for lncRNAs [103] and verified the interaction between lncRNAs and other biomolecules [104], respectively.

## 7. Techniques and Methods of LncRNAs Study in *Drosophila*

Research on lncRNAs in *Drosophila* mainly focuses on the identification, function verification, and mechanism exploration. To precisely characterise their mechanisms of action, a large number of techniques in sequencing, bioinformatics analyses, and experimental verifications have been established and widely used.

### 7.1. LncRNA Identification

Modern techniques such as microarrays [78] and RNA sequencing [55,56] provide a high-throughput approach by which the *Drosophila* lncRNAs expression can be detected on a large scale. Besides, since lncRNAs expressed in only a minority of cells may be undetectable, single-cell-sequencing is developed to remove this barrier by assessing gene expression within individual cells. For example, Drop-seq could output transcriptome at single-cell resolution [63,65], which allows for gaining novel insights into the cell-to-cell heterogeneity and the sensitivity of lncRNA detection. With Northern-blot [12] and qRT-PCR [75], the authenticity of sequencing results and lncRNA expression levels can be tested. To investigate possible functions of novel lncRNAs, the full sequence needs to be determined, for which 5′ and 3′ rapid amplification of cDNA ends (RACE) are performed [73]. Bioinformatics analysis is an accurate and convenient approach that can rapidly generate helpful information for further verification [113], as coding potential could be predicted by software. In vitro translation assay [76] could further confirm the non-protein-coding capacity of lncRNAs. However, these techniques still have some limitations. For RNA-seq, cDNA synthesis is not suitable to analyse short RNAs, degraded and/or small quantity RNA samples [114], while lncRNA microarray could only detect those previously described candidates that are already in the lncRNA-related database. qRT-PCR exhibits a disadvantage of high cost but with a low throughput [115].

### 7.2. Function Verification

Two principal manipulations are usually employed to study gene functions, which are loss-of-function (LOF) and gain-of-function (GOF) [9]. Both of them can be achieved in *Drosophila* with the help of RNAi [75,78], gene targeting [81], the CRISPR/Cas9 system [56,116], and targeted over-expression of genes [18,73]. Employment of the versatile Gal4/UAS system enables genes to be regulated in a tissue- and temporal-specific manner and makes rescue analysis possible [81]. Moreover, since the function of lncRNAs is closely linked to their unique subcellular localization patterns, RNA in situ hybridization [70,77] based on molecular hybridization is a well-established method to determine their localization and visualize their expression patterns. However, limitations still exist. RNAi is effective for cytoplasmic lncRNAs but is relatively inefficient for lncRNAs localized in the nucleus, which could be attributed to the fact that some nucleus-reserved lncRNAs might be insensitive to short hairpin RNAs (shRNAs) or siRNAs. In addition, the existence of negative feedback regulation could also cause some problems. Thus the application of RNAi may only lead to a decline, rather than an elimination of the lncRNA function.

### 7.3. Mechanism Exploration

Since altering activity of promoters is a major way for lncRNAs to regulate target genes transcription, several techniques aimed at this mechanism have been developed. For example, luciferase assays have been performed to examine whether lncRNA-CRG regulates *CASK* transcription through its alteration of the *CASK* promoter regions [76]. For the dissection of promoter regions and the investigation of expression patterns of lncRNAs, a GFP transformation system could be utilized [79]. Interactions between promoters and certain proteins could be verified by CHIP [73,76]. Electrophoretic mobility shift assay (EMSA) [12] and RIP [73] can be used for verifying protein–lncRNA interactions. The application of chromatin isolation by RNA purification (ChIRP) to lncRNA-roX2 studies shows that it indeed binds hundreds of places on the *Drosophila* X-chromosome, whose locations correlate perfectly with binding sites of the protein cofactor MSL2 [117]. Moreover, RNase footprinting and selective 2′-hydroxyl acylation analyzed by primer extension (SHAPE) chemistry have been used to explore how lncRNA-roX1 and roX2 coordinate the binding of chromatin-modifying proteins to control dosage compensation [118]. The drawback of enzymatic footprinting and chemical probing technique is that enzymatic footprinting cannot be applied in vivo and it cannot identify specific base pairs, which might lead to incorrectly predicted secondary structures [119].

The numerous methods discussed above make it possible to uncover novel lncRNA functions at a fast pace. More and more information on sequencing is generated, although most of it is under-used [114]; therefore, novel and effective computational strategies need to be developed to exploit the underlying value of that information. At the same time, improvements in existing techniques are also needed to overcome those limitations. The development of new technology will help us further decode the function and regulatory mechanisms of lncRNAs.

## 8. Conclusions and Future Perspectives

Numerous lncRNAs have been discovered through the development and application of high-throughput transcriptome sequencing, and have gradually emerged as crucial biological function participators in in-depth studies. With high spatiotemporal specificity and preserved secondary structures, the patterns of action of lncRNAs in biological processes appear to be more diversified. Mechanisms of lncRNAs in regulating gene expression are accomplished through the modification, stabilization, and translation of mRNAs. In addition, lncRNAs account for a significant proportion of the human genome and act throughout various life activities, including growth, development, ageing, and death. Much evidence shows that lncRNAs can be regarded as effective diagnostic and prognostic molecular hallmarks and potential therapeutic targets in diseases such as cancers and cardiovascular pathologies. Besides, the improvement of survival prognosis after infection by overexpressing lincRNA-IBIN hints at the possibility of utilizing *Drosophila* to analyse the side-effects of lncRNA therapy [18].

*Drosophila* is an ideal animal model for exploring the molecular mechanisms of organism development and multiple human diseases such as cardiovascular disease, cancer, and various neurological diseases. Transcriptomics analysis exhibits a main connection between lncRNAs and gonads and development, while lncRNAs directly participate in embryo development, neurological function, gonadal function, and anti-stress ability, and have been validated with experimental verification. In addition, given the high similarity between certain regions of the lncRNA-7SK sequence in *Drosophila* and that in humans, and the very similar lncRNA-7SK-based transcriptional regulatory system that exists in both [12], it is reasonable to infer that the mechanisms found in *Drosophila* may be carried forward into other organisms and humans. Therefore, *Drosophila* can be regarded as an expanding arena for researching the mechanisms of lncRNAs. Mechanisms of lncRNAs in humanized flies and some disease models need to be explored in the future. Bioinformatics analysis of spatiotemporal-specific patterns and secondary structure information may provide an appropriate direction to recognize conserved lncRNAs.

## Figures and Tables

**Table 1:** LncRNAs in *Drosophila*.

Transcripts	Related Genes/Proteins	Function	Reference
AAGAG repeat RNAs	-	Nuclear matrix constituents	[70]
acal	*Cka*, *aop*, *raw*	Involved in dorsal closure	[68]
ASTR	*regena*, *sisR-1*	Regulates *regena* gene transcripts	[69]
bereft	*cut*, *numb*, *tramtrack*	Involved in bristle morphogenesis	[72]
bxd	*Ubx*	Regulates growth and development	[13]
CRG	*CASK*, RNA pol II	Regulates locomotor activity and climbing ability	[76]
CR18854	*dFUS*, *dFIG4*	Suppresses the rough eye and the loss-of-cone cell phenotype caused by *dFIG4* deficiency	[15]
hsrω	hnRNPs, HP1, RNA pol II	Responds to heat shock	[17]
hsrω	*dFUS*	Regulates the development of neuromuscular junctions	[14]
iab-8	*miR-iab-8*, *abd-A*	Regulates the mating behaviour	[77]
IBIN	Toll, BAP, Brahma	Acts as a link between innate immune responses and metabolism	[18]
lincX	*Scr*	Involved in the activation of *Scr*	[67]
msa	*miR-iab-8*	Involved in accessory gland development and male fertility	[16]
oskar	Bruno, Staufen	Regulates oogenesis	[74]
roX1 and roX2	Msl, Mof, Mle	Involved in dosage compensation	[78]
SMRG	*scute*, E(spl)mβ	Regulates scutellar macrochaetes	[73]
sphinx	-	Involved in the regulation of male courtship behaviour	[79]
Sxl_Pe_-R1 and R2	*Sxl*, *PcG/trxG*	Facilitates sex determination	[80]
vg-PRE/TRE	*vg*, PRC2, E(Z)	Regulates PRC2 activity	[71]
yar	-	Regulates sleep behaviour	[81]
7SK	RNA pol II, P-TEFb	Regulates RNA pol II activity via P-TEFb	[12]

“-” means no report.

**Table 2:** *Drosophila* lncRNA-related databases.

Name	Website	Description	Reference
FlyBase	http://flybase.org	A database of *Drosophila* genes and genomes	[105]
FlyAtlas 2	http://www.flyatlas2.org	Gene expression pattern in fly tissues	[101]
DVEX	http://www.dvex.org	Single-cell expression atlas of lncRNAs of the stage 6 *Drosophila*	[63]
DroID	http://droidb.org/	Interaction networks (protein–protein, TF–gene, and miRNA–gene)	[102]
NONCODE	http://www.noncode.org/	Details of annotation of lncRNAs	[106]
lncRNAdb	http://www.lncrnadb.org/	Information on RNAs related to nucleotide sequence, genomic context, gene expression data, structural information, subcellular localization, conservation, and function (validated data)	[107]
LncVar	http://bioinfo.ibp.ac.cn/LncVar/	Systematically integrated information about transcription factor binding sites and m6A modification sites of lncRNAs and comprehensive effects of single nucleotide polymorphism (SNPs) on transcription and modification of lncRNAs	[108]
LNCediting	http://bioinfo.life.hust.edu.cn/LNCediting/	A comprehensive resource for the functional prediction of RNAs editing in lncRNAs	[109]
ChIPBase v2.0	http://rna.sysu.edu.cn/chipbase/	Transcriptional regulatory networks of non-coding RNAs (ncRNAs) and protein-coding genes (PCGs)	[110]
CRISPRlnc	http://www.crisprlnc.org	Manually curated database of validated CRISPR/Cas9 sgRNAs for lncRNAs	[103]
NPInter v3.0	http://www.bioinfo.org/NPInter/	Experimentally verified interactions between ncRNAs (excluding transfer RNAs (tRNAs) and ribosomal RNAs (rRNAs)), especially lncRNAs and other biomolecules	[104]
CLIPdb	http://lulab.life.tsinghua.edu.cn/clipdb/	Regulatory networks among RNA-binding proteins and various RNA transcripts	[111]
lncRNAtor	http://lncrnator.ewha.ac.kr/	Information related to expression profiles, interacting (binding) proteins, integrated sequence curation, evolutionary scores and coding potential of lncRNAs	[112]
